# Education, Age and Gender: Critical Factors in Determining Interventions for Child Brick Workers in Pakistan and Afghanistan

**DOI:** 10.3390/ijerph19116797

**Published:** 2022-06-02

**Authors:** Catherine Pellenq, Laurent Lima, Susan Gunn

**Affiliations:** 1 Laboratoire de Recherche sur les Apprentissages en Contexte/Research Laboratory on Learnings in Context, Grenoble-Alpes University, LARAC/1251 Avenue Centrale, Domaine Universitaire, 38000 Grenoble, France; laurent.lima@univ-grenoble-alpes.fr; 2 International Labour Organisation, 1211 Geneva, Switzerland; suzanegunn2@gmail.com

**Keywords:** child labor, gender, age, psychosocial well-being, health, brick kilns, Pakistan, Afghanistan

## Abstract

Working in factories fashioning bricks by hand seems the epitome of hazardous child labor. Yet, efforts to remove children from this work have shown little success; impoverished families balance the value of their children’s contribution against the risks they see. Unfortunately, psychosocial impacts are often not visible, and are rarely taken into consideration when designing interventions. A comprehensive occupational health study of children working in brick factories included a module on psychosocial risks and impacts. This analysis reports on the Pakistan and Afghanistan portion of the study which was administered to 450 child brick workers and 486 controls, aged 11–17. Factorial ANOVAs confirmed that working in brick factories was the strongest predictor of respondent’s psychosocial health. However, they also identified subgroups of children that escape this prediction. Older girls, for example, actually felt better when working, compared with staying at home. Schooling had positive associations, especially in younger boys and adolescent girls. In fact, the results of this study showed that those who are at greatest psychosocial risk were girls who do not go to school. These findings underscore the importance of assessing psychosocial impacts and tailoring policy and interventions to specific gender and age categories of young workers.

## 1. Introduction

For child workers, the risks and impacts on their physical health and education are relatively well-documented, but how does work affect children’s psychological health? Working children include both those who work legally and those who are classed as ‘child labor’ by virtue of their age or the risks they face, yet we know little as to which working children—younger or older, girls or boys—are at greater risk, nor do we know the extent to which other factors such as schooling may act to augment or mitigate the risk. 

Although researchers increasingly recognize the importance of understanding children’s own perspectives on their lives, it is still rare for comparative studies to include quantitative measures of children’s subjective well-being [1,2,3]. This is especially true for studies of working children in spite of systematic reviews expressing concern about this omission [4,5,6,7,8,9], and the fact that “children in employment” constitute a very significant segment of the total child population (222,088,000, aged 5–17, are estimated to be economically active) [10]. This study joins a small group of national studies which have been undertaken in what is proving to be an extremely important aspect of child workers’ well-being [11,12,13].

The analysis presented in this paper draws on a dataset from a multi-country occupational health study of 1556 children working in brick factories [14,15,16,17,18]. The study sought to demonstrate a methodology for identifying the categories of child workers which are most likely to need urgent protection and intervention, a requirement for all countries under ILO Convention No. 182 on the Worst Forms of Child Labour. 

Brick manufacture was selected because it is a global industry with a significant percentage of child labor (20% to even 45% in some cases). Employing an estimated 20 million workers in over 20 countries, it has been increasing to meet the demand for building materials for the rapidly growing cities of Asia, Africa, and South America. In those factories where bricks are fashioned by hand using traditional methods, children are engaged to perform such simple but essential tasks as turning the raw bricks so they dry evenly. Because the children’s help increases the efficiency of the adults, and because the families who work in the factories are some of society’s most disadvantaged who have few other options for employment, it is not a simple matter for them to release the children from work in spite of laws to that effect. It is not surprising that efforts to reduce child labor in the brick industry have met with little success. 

While parents and employers may view children’s work in the brick factories as logical, practical, and relatively safe, child workers may have a different perspective. However, other than anecdotally, the feelings they have about their lives and future are generally not solicited. Little is known about the emotional and intellectual effects, both immediate and longer term, of doing dull repetitive work or of working in harsh or stressful conditions in this industry and others like it. 

To address this lack, it was necessary to develop an instrument that was relevant from three perspectives, that of children, work, and psycho-social well-being. The new instrument was grounded in the pioneering theoretical work of Martin Woodhead (2004) [19] on the psychosocial factors relevant to children’s work. It then drew on a wide range of existing validated pediatric, psychological, and occupational health assessments to select items within the domains he had posited as most relevant. The final result was subjected to repeated validations [20,21]. 

When the larger occupational health study was completed, the results from the psychosocial component were found to be particularly intriguing. They showed significant differences between the psychosocial profiles of children who worked in the brick factories and those who have never done such work [22]. The child workers evidenced significantly greater psychological stress, insecurity, and negative emotions (e.g., fear, hostility) but none of the ameliorating factors that work with family was expected to have. There was also evidence that girls were experiencing greater psychosocial impacts [17,18]. 

To see how personal or contextual factors might be interacting to influence or impact the psychosocial health of the working children, we undertook a new analysis of the data from two countries—Pakistan and Afghanistan—which were more homogeneous with regard to cultural, religious, and linguistic background than the larger study. The specific aim was to understand to what extent the psychological burdens and benefits are borne equally by boys and girls, whether age or education are factors in how the child workers cope with the psychosocial stresses associated with their jobs, and whether there are significant interactions between work, gender, and age as suggested by some studies [23,24]. This paper reports on the results of this analysis.

## 2. Material and Method

This analysis extracts data from a comparative population-based occupational health study in Afghanistan and Pakistan and compares psychosocial data from 936 children (450 child brick workers and 486 children who do not do this work) aged 11–17. It uses Factorial ANOVAs to examine the simple effects and the interactions of four variables—work (in brick factories), age, gender, and education—on the subjective psychosocial well-being of children. 

### 2.1. Study Population

This analysis draws on data from five randomly selected brick manufacturing sites in the Pakistan provinces of Punjab, Sindh and Khyber Pakhunkhawa and two sites in the Afghan provinces of Kabul and Nangarhar.

The study population comprised children who were currently residing and working at the brick factories, and a comparison group from nearby communities who had never worked in the brick industry. To the degree possible, the comparison group was matched to the workers with respect to key socio-demographic characteristics such as age, ethnic and linguistic background, and socio-economic strata as evidenced by local indices of wealth. 

The respondents were randomly selected from a site-based census and consisted of 1130 girls and boys between 11 and 18 years of age; 936 had valid data. We compared two age groups, the first consisting of children under 14 years of age, which is the age limit set in both Afghanistan and Pakistan defining child labor as per ILO Convention No.138, and a group of older children aged 14–17 years (Table 1). As some working children attend school while others do not, four groups of children were compared: (a) working children who attend school, (b) working children who are not attending school, (c) children from the comparison group who attend school, and (d) comparison children who do not attend school. In doing so, we control for the combined effects of working and attending school of older children aged 14–17 years which could be considered regular legal employment if the tasks or conditions are not demonstrated to be hazardous.

### 2.2. Procedure

Ethical clearance was obtained through generally accepted procedures in the two host countries, consent was sought from children, as well as from their parents and from the employers of the respondents. 

All children, from both the study population and comparison group, were interviewed by specially trained interviewers. The interviews included 4 questionnaires on: (1) socio-demographic characteristics (age, sex, household composition, attendance, and level of schooling), (2) work history (task list, starting age of work, hours of work per day and week, other work, household chores, work in the off-season), (3) physical health assessment (history of injuries, illnesses, recent health incidents, sleep, fatigue), (4) psychological health assessment using the Instrument for Psychosocial Assessment of Child Workers [20,21]. This paper draws on data from questionnaires 1 and 4 (for a comprehensive report of the four-country study see [14]).

The child worker interviews were conducted at the work site during a break and within sight of parents or guardians especially in the case of girls; the comparison groups were interviewed in the school environment. In conformity with local custom, girls were interviewed by female interviewers who spoke the same dialect as the respondents. The children were not paid, but in the case of Pakistan were given vitamin/mineral supplements which were appreciated.

### 2.3. Material

The IPAW questionnaire was developed and validated to serve as a measure of child workers’ subjective well-being [21]. It consists of 48 items, divided into two parts: the first concerns life at work and is administered only to working children; the second part concerns life outside the work context and is administered to both working children and the comparison group. 

**Figure 1:**
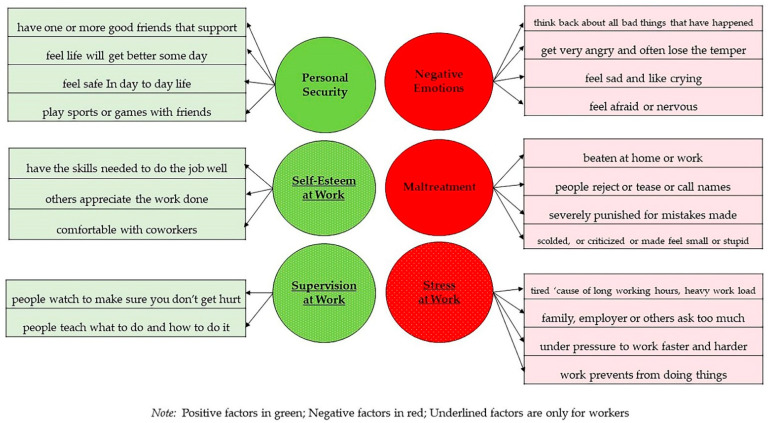
IPAW’s factorial structure (adapted with permission from Pellenq et al., 2021 [18]).

The items cluster around 6 dimensions, closely following the typology established by Martin Woodhead in his seminal paper on child labor and work [19]. They include: i. self-esteem at work; ii. supervision at work; iii. stress at work; iv. feelings of personal security; v. negative emotions (such as fear and anger); vi. feelings of abuse and mistreatment (Figure 1).

### 2.4. Statistical Analysis

Factorial ANOVAs were run with SPSS software, taking into account the variables Work, School, Age, and Gender, on the three dimensions of psychosocial well-being: Negative Emotions, Personal Security, and sense of Mistreatment. The statistical outputs can be found in the Supplementary Material file.

## 3. Results

On each of the 3 factors used to compare workers and controls, the effects of working in kilns, being aged between 11 and 13 years old or 14 and 18 years old, being a girl or a boy, and attending school or not, are measured. Table 2 provides a description for these factors by age group. Results showed that the working children had unique psychosocial characteristics when compared with those who did not work.

### 3.1. Children Who Often Express Negative Emotions 

The Negative Emotions dimension includes items related to internalized or externalized negative emotions such as rumination, sadness, anger, and fear. It is an indicator of depression. On it, we investigated the effect of working in kilns (variable ‘work’), of being a girl (variable ‘gender’), of belonging to a younger or older group (variable ‘age’), and of attending school or not (variable +/− ‘school’).

The model had an explicative power of 18% (estimated with the coefficient R^2^). The simple effect of Work—whose size is estimated with eta-squared (η^2^)—was more powerful (*F*(3, 933) = 8.33, *p* < 0.001, η^2^ = 0.027). It indicated that working children display many more Negative Emotions than those in the comparison group. Then the following simple effect, to a lesser degree, was that of Gender (*F*(1, 933) = 8.67, *p* = 0.003, η^2^ = 0.009): girls had higher scores of negative emotions than boys; they felt more depressed. Age presented a non-significant effect: the degree of expressed depression was similar among both younger and older children. 

**Figure 2:**
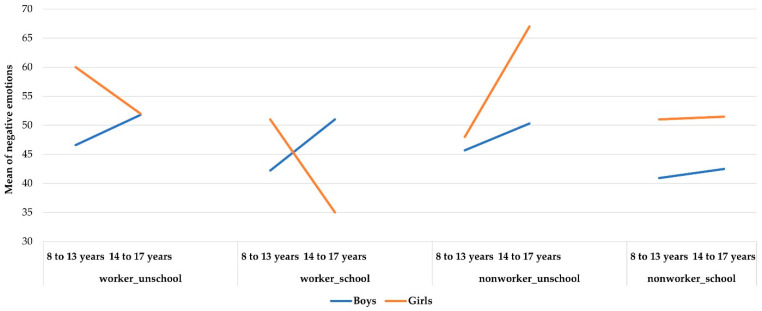
Gender differences for Negative Emotions.

Although these results were generally consistent with what was hypothesized based on results from the larger four-country sample, the analysis displayed complex interactions that were unexpected (Figure 2). There was a significant interaction between Work and Age (*F*(3, 933) = 4.58, *p* = 0.003, η^2^ = 0.015) and a double significant interaction between Work, Age, and Gender (*F*(3, 933) = 5.33, *p* = 0.001) with rather identical size effects (respectively, η^2^ of 0.015 and 0.017). These results indicated that males and females were affected differently by work if they were in the cohorts above or below 14 years of age. The graphs below plot these combined effects.

These graphs show the interaction effect between Work +/− School and Age for boys and girls on Negative Emotions.

For boys, the worst situation is not going to school, independent of whether they work or not. Boys who did not go to school showed higher levels of negative emotions than those boys who attended school. This effect was even more pronounced in the older age group. The best situation for boys was to not work and to go to school, regardless of age.

For younger children, working and going to school was a favorable situation. Their scores were almost at the same level as those in the non-working comparison group who are in school. In effect, school removed the negative effect of work among younger children. However, it is the opposite for older working children: schooling did not ameliorate the depressive emotional states of adolescents. School had a protective effect on depression only on younger working children. These findings suggest that, for adolescents, school or more particularly the type of schooling available to them, seems to present them with emotional challenges. 

For girls, the emotional situation is much more complicated. Depression scores were higher than those of boys and interactions were more complex. Like boys, the younger girls were in an unfavorable situation when working without going to school but the three other conditions were equivalent, none was better or worse. It was different for older girls: for them, the more unfavorable situation was to not work and not go to school (like boys), and the more favorable was to work and to go to school (whereas for boys it is unfavorable). In other words, the environment which they find more supportive is not the home but rather outside it, either working or going to school. What is worst for them was to neither work nor go to school. Culture may be a factor here. Girls in mid-adolescence may have the opportunity for more social interactions in the work environment than in the home environment as of that age, or the home environment may entail more strenuous work, greater likelihood of early marriage, or reduced sense of safety. 

### 3.2. Children Who Feel Mistreated at Work or at Home 

The Maltreatment factor includes items concerned with being beaten or punished, being criticized, and feeling rejected. The model had an explicative value of 13%. The more significant effect on maltreatment was that of Work +/− School (η^2^ = 0.055), with a smaller effect of Age (η^2^ = 0.009). This indicated that working children and younger children felt more maltreated than, respectively, the non-working children and the older children. There was a non-significant simple effect of Gender. There were an interaction between Gender and Age (*F*(3, 935) = 4.96, *p* = 0.026, η^2^ = 0.005), between Gender and Work +/− School (*F*(3, 935) = 2.71, *p* = 0.044, η^2^ = 0.009, and a double interaction effect between Gender, Age, and Work +/− School (F(3, 935) = 4.96, *p* = 0.034, η^2^ = 0.009.

**Figure 3:**
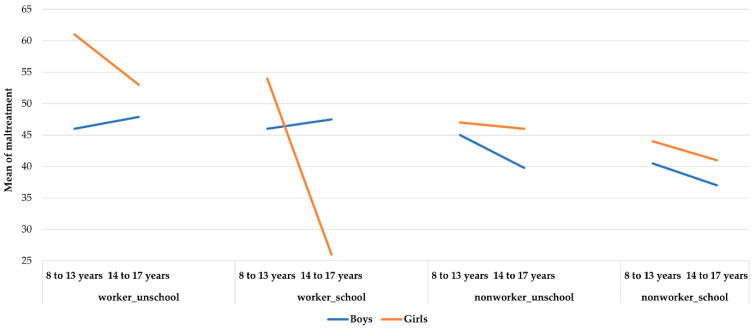
Gender differences for Mistreatment.

The graphs (Figure 3) show the interaction effect between Work +/− School and Age for boys and for girls on sense of being mistreated. 

Boys who felt more maltreated were those who work, regardless of whether they went to school or not; school did not reduce the working boys’ sense of maltreatment. It is interesting to note that non-working boys who did not attend school had a similar expression. The more favorable situation for younger boys was to go to school full time. For this younger age group, working was not worse than doing nothing or staying at home; they found it better to go to school or to work. This suggests that there may be dynamics operating in the home environment in this cultural context that bear further inquiry. 

For older boys, the fact of working was always determinative (negative), regardless of whether or not they attended school. For girls, the situation was different than that of boys and was what we expected to find. There was a small protective effect of being at school for younger working girls. The most favorable situation was to attend school full time. The worst was to work full time. The worst situation for older girls was to work full time and the best one was to attend school and to work. Those who do not work were in an intermediate position. 

### 3.3. Children Who Express a Weak Feeling of Security 

The Security factor draws on items in the questionnaire on the feeling of personal safety, support from friends and family, and hope for a better life.

The model had a higher R2 index (0.47) and gave a large Work +/− School effect of 0.358. Children who work had a much lower sense of security than those who did not work. This indicated that Work strongly affects the sense of personal security, regardless of age or gender. There was a smaller significant simple effect of Gender (η^2^ = 0.012) with girls feeling less secure than boys. However, there were significant interaction effects between Work +/− School and Gender (*F*(3, 933) = 4.61, *p* = 0.003, η^2^ = 0.015) and of Work+/− School and Age (*F*(3, 933) = 3.73, *p* = 0.001, η^2^= 0.012).

**Figure 4:**
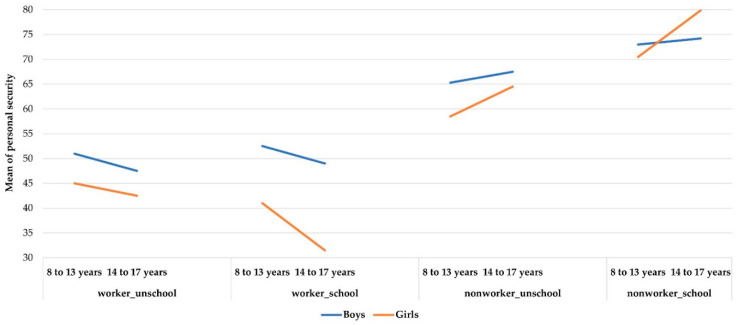
Gender differences for Personal Security.

These graphs (Figure 4) show the interaction effect between Work +/− School and Age for boys or for girls on Personal Security. Non-workers had consistently higher scores on this factor than did workers, regardless of gender. There was a difference only among older children between working girls and boys as to attending school or not. Girls who work and attend school, especially those 14–17 years old were more insecure (there was no difference for non-workers, who were parallel). For boys, what was determining was whether they were working or not. For girls, it was the same but attending school was also important. 

## 4. Discussion

We analyzed several factors which affect the sense of well-being for approximately 1000 children living in proximity to brick factories in Afghanistan and Pakistan. Work in the brick factories was ascertained to be the strongest predictor on all dimensions of well-being (it had a positive effect on negative factors, and a negative effect on positive factors). It accounted for the largest effect size: very strong and negative on the feeling of personal security which is related to family and social support, and hope for a better life (0.358), and positive on the feeling of abuse (0.055) and on experiencing negative emotions—(0.027). The findings from this analysis corroborate those obtained by Pellenq et al. (2021) [18] on a larger, more heterogeneous sample, namely: the hypothesis of a causal relationship between work and the deterioration of psychological health is plausible. 

This analysis presents a more nuanced understanding of the nature and severity of the overall psychological impacts for children working in a brick factory. It demonstrates that it is not necessarily the youngest children, as is often supposed, who are at greatest risk. Rather, it was frequently the adolescent workers, in the age group 14–17 years, who expressed significantly more negative emotions than younger children.

The fact that older children are reflecting a more negative experience invites further study. They may be reacting to the constraints posed by authority or circumstance, or they may be more aware than younger children of limited options for the future. It is known that high sensitivity to stress is a characteristic of adolescence [25] and exposure to hazardous work may be exacerbating this.

What then does schooling bring to these young workers? While it appears that attending school is almost always beneficial for working children, its protective value depends on the psychosocial factor considered, as well as the gender and the age of the worker. Except for older boys, attending school is protective against depression, a little less protective on the feeling of abuse, but has no positive effect on the feeling of personal security. A striking fact from this analysis is that attending school—at least in this cultural context—can be negative in a subcategory of children, here on the feeling of safety of the oldest working girls. This effect does not exist for boys.

Limitations: The explanatory power of the models tends to be weak. This may be due to large intra-group dispersion, such as variation in whether the child workers have migrated from outside the area, live and work alone or with parents and/or siblings, are free or bonded labor, etc. There are also probably important local effects such as conflict or instability in the region, availability and distance from schools. Furthermore, the data on school enrolment, attendance, completion, and quality of instruction are not precise, or in some cases missing altogether. 

In the case of this study, a quantitative approach was considered necessary in order to determine broad characteristics and differences among populations, as well as to model a better balance between physical and psychological areas of inquiry which, as noted above, is severely wanting in studies of this nature. Qualitative methods, capable of soliciting individuals’ views, are essential for exploring, explaining, and confirming the differences which have been highlighted by the quantitative component; for example, the factors behind negative psychosocial feelings and why expected prediction fails in some categories of children. (An additional qualitative component to examine reasons for psychosocial malaise of working girls was halted due to the pandemic and the unstable situation in the countries studied.) 

Further research: These findings support the call for further research into the differential gender effects of work on children, especially with regard to education. It is crucial to understand what factors in the school environment are protective and which constitute challenges to child workers’ well-being; this is especially important given that education is the primary intervention for addressing child labor. Further research could also examine the data on child workers from the standpoint of all six well-being factors, not just half, which would lead to a more detailed psychosocial profile relative to work. A longitudinal perspective would enable us to relate physical and psychosocial well-being to the starting age and number of years children have been working in the brick factories. Including young adults (18–24 years old) in the sample could allow a first estimate of the medium-term impact of early entry into work. Finally, as noted above, qualitative data are needed to understand the mechanisms that are at the origin of the differences between groups of children.

## 5. Conclusions

These findings lead us to conclude that, in addition to potential physical risks, the conditions in which children work in the brick factories impose psychological risks as well. On the whole, the situation is clearly not conducive to psychological well-being, contrary to our expectation that the work would produce at least some psychosocial benefits (e.g., contribution to family welfare, self-efficacy). Sense of security has emerged as an important variable as in the case of older girls for whom the work situation may be ‘less bad’ than the alternatives they have available. 

Overall, this study shows that children working in brick kilns today are paying a high price in terms of their psychological health and education, and will likely perpetuate the cycle of poverty in which their families are entrenched. Compared to their peers who do not work in the brick factories, they feel more depressed, anxious, and afraid; they feel more mistreated and at risk of being criticized, punished, or beaten. Even worse, they do not feel they are supported by their families or peers, which contributes to a general sense of insecurity and lack of hope for improvement in their lives in future. Especially alarming is the fact that a significant proportion of the workforce in brick factories accumulates multiple disadvantages: that of being a child, that of being a worker, and that of being a girl. It is girls who pay the highest price. 

The findings give us not only the incentive to intervene but also indications as to the kind of interventions needed. First, it argues against a broad-brush approach that treats all child workers the same regardless of age or gender in favor of one that identifies and targets sub-groups in the child-worker population. Second, the interventions must be designed, not as general poverty alleviation programs, essential as these are, but from a specifically child-centered and culturally sensitive perspective. What may make sense for the majority of the population can actually have negative consequences for child workers; special care needs to be taken in how labor laws are enforced, for example. Third, these results provide justification for vastly expanded educational opportunities for all age groups, especially in rural areas. While issues of quality of education have long been emphasized, these results underscore that security in the school environment is of great, maybe even greater importance in this context. Especially for older girls, a safe and supportive school environment is crucial.

The value of a study of this kind is that it provides information for families who must weigh the advantages of work against its risks. However, when conveying information about negative effects, care must be taken so as not to ignore or undermine the efforts they are already making to protect their children, or to undermine the child workers’ pride in the work they do. Employers and unions would find this information helpful when assigning appropriate and safe tasks to children of working age, and political decision-makers can take psychosocial information into account when considering what to include in the list of hazardous child labor to be prohibited to children under age 18 as required by ILO Convention No. 182.

Finally, in demonstrating that work has influential and differential effects on children’s sense of well-being, this study adds further support to the argument that the views of the children themselves must be taken into account, both in understanding the nature of the problems they face and in the interventions to be taken to address these problems.

## Figures and Tables

**Table 1:** Variables and frequencies.

Between Subject Variables	Sub-Categories	*n*
Gender	Boys	699
	Girls	237
Age	8–13 years old	454
	14–17 years old	482
Work and School	Workers no-school	383
	Workers + school	68
	Controls no-school	132
	Controls + school	345

**Table 2:** Mean and standard deviation of each of the 3 factors of well-being, by age groups.

		Workers	Controls
Negative emotions	11 to 13 years old	48.26 (16.68)	48.78 (13.78)
	14 to 17 years old	50.07 (16.12)	49.52 (18.04)
Maltreatment	11 to 13 years old	48.20 (17.34)	45.07 (12.41)
	14 to 17 years old	45.62 (17.83)	43.91 (11.03)
Personal security	11 to 13 years old	49.31 (13.80)	70.22 (12.80)
	14 to 17 years old	47.48 (14.70)	71.70 (12.55)

## Data Availability

The data on which the analysis in this study rest are available on reasonable request from the corresponding author, although the primary data are restricted due to the fact they pertain to children and are governed by the countries in which the research was undertaken.

## Supplementary Materials

The following supporting information can be downloaded at: https://www.mdpi.com/article/10.3390/ijerph19116797/s1, tables of factorial anovas (adapted from SPSS output files) testing for between subjects effects.
