# Supplementary material for: Education, Age and Gender: Critical Factors in Determining Interventions for Child Brick Workers in Pakistan and Afghanistan
Source: Int J Environ Res Public Health. 2022 Jun 2;19(11):6797. doi: 10.3390/ijerph19116797 (PMC9180764; doi:10.3390/ijerph19116797)
Supplement: Supplementary file 1 [file ijerph-19-06797-s001.zip › ijerph-1706876-supplementary.pdf]

Education, Age and Gender: Critical Factors in Determining Interventions for Child Brick Workers in Pakistan and Afghanistan

Catherine Pellenq, Laurent Lima and Susan Gunn

Supplementary Material: Tables of factorial ANOVAs (adapted from SPSS output files) testing for between subjects effects.

Section S1: Tables for the factor «negative emotions»
Section S2: Tables for the factor «maltreatment»
Section S3: Tables for the factor «personal security»

Section S1: Tables for the factor «negative emotions»

Factors		values	N
Gender Female	0.00		699
	1.00		235
Age category	1.00	8 to 13 years old	453
	2.00	14 to 17 years old	481
Work & school	1.00	Worker no school	383
	2.00	Worker + school	67
	3.00	nonworker_no school	130
	4.00	Nonworker +school	354

Tests of beetwen-subjects effets

Dependant variable: negative emotions

	Sum of squares type III	ddl	Mean square	F	Signification	Eta-squared (size effect)
Corrected model	44,957.474	15	2997.165	13.299	0.000	0.179
Constant	598,170.097	1	598,170.097	2654.226	0.000	0.743
female	1955.125	1	1955.125	8.675	0.003	0.009
age category	243.862	1	243,862	1.082	0.299	0.001
Works&school	5633750	3	1877.917	8.333	0.000	0.027
female * age category	590,370	1	590,370	2.620	0.106	0.003
female * travailecole	1062.018	3	354,006	1.571	0.195	0.005
age category * Works&school	3094.071	3	1031.357	4.576	0.003	0.015
female * age category * Works&school	3604.251	3	1201.417	5.331	0.001	0.017
Error	206,885.239	918	225.365			
Total	2,463,515.625	934				
Corrected total	251,842.713	933				

R-squared = 0.179 (adjusted R-squared =0.165)

Section S2: Tables for the factor « maltreatment »

Factors		values	N
Gender Female	0.00		699
	1.00		237
Age category	1.00	8 to 13 years old	454
	2.00	14 to 17 years old	482
Work&school	1.00	Worker no school	383
	2.00	Worker + school	67
	3.00	nonworker_no school	132
	4.00	Nonworker +school	354

Tests of beetwen-subjects effets

Dependant variable : maltreatment

	Sum of squares type III	ddl	Mean square	F	Signification	Eta-squared (size effect)
Corrected model	33,790.695	15	2252.713	9.485	0.000	0.134
Constant	489,566.544	1	489,566.544	2061.371	0.000	0.691
female	370.319	1	370.319	1.559	0.212	0.002
age category	1991.647	1	1991.647	8.386	0.004	0.009
Works&school	12,810.142	3	4270.047	17.979	0.000	0.055
female * agecategory	1177.176	1	1177.176	4.957	0.026	0.005
female * Works&school	1930.019	3	643.340	2.709	0.044	0.009
agecategory * Works&school	528.525	3	176.175	0.742	0.527	0.002
female * agecategory * Works&school	2062.554	3	687.518	2.895	0.034	0.009
Error	218,495.922	920	237.496			
Total	2,124,023.438	936				
Corrected total	252,286.617	935				

R-squared = 0.134 (adjusted R- squared = 0.120)

Section S3: Tables for the factor « personal security »

Factors		values	N
Gender Female	0.00		697
	1.00		237
Age category	1.00	8 to 13 years old	452
	2.00	14 to 17 years old	482
Work&school	1.00	Worker no school	381
	2.00	Worker + school	68
	3.00	nonworker_no school	131
	4.00	Nonworker +school	354

Tests of beetwen-subjects effets

Dependant variable : personal security

	Sum of squares type III	ddl	Mean square	F	Signification	Eta-squared (size effect)
Corrected model	133,388.208	15	8892.547	54.797	0.000	0.472
Constant	786,560.211	1	786,560.211	4846.864	0.000	0.841
female	1808.109	1	1808.109	11.142	0.001	0.012
age category	2.323	1	2.323	0.014	0.905	0.000
Work&school	83,226.661	3	27,742.220	170.950	0.000	0.358
female * agecategory	38.299	1	38.299	0.236	0.627	0.000
female * Works&school	2243.563	3	747.854	4.608	0.003	0.015
agecategory * Work&school	1815.101	3	605.034	3.728	0.011	0.012
female * agecategory * Work&school	409.810	3	136.603	0.842	0.471	0.003
Error	148,975.157	918	162.282			
Total	3,669,257.813	934				
Corrected total	282,363.365	933				

R-squared = 0.472 (adjusted R-squared = 0.464)
